# Metabolic Flexibility in Response to Within-Season Temperature Variability in House Sparrows

**DOI:** 10.1093/iob/obaa039

**Published:** 2020-11-05

**Authors:** D L Swanson, T J Agin, Y Zhang, P Oboikovitz, S DuBay

**Affiliations:** 1 Department of Biology, University of South Dakota, Vermillion, SD 57069, USA; 2 Department of Ecology and Evolutionary Biology, University of Michigan, Ann Arbor, MI, USA

## Abstract

The climatic variability hypothesis (CVH) posits that more flexible phenotypes should provide a fitness advantage for organisms experiencing more variable climates. While typically applied across geographically separated populations, whether this principle applies across seasons or other conditions (e.g., open vs. sheltered habitats) which differ in climatic variability remains essentially unstudied. In north-temperate climates, climatic variability in winter usually exceeds that in summer, so extending the CVH to within-population seasonal variation predicts that winter phenotypes should be more flexible than summer phenotypes. We tested this prediction of the within-season extension of the CVH by acclimating summer and winter-collected house sparrows (*Passer domesticus*) to 24, 5, and −10°C and measuring basal metabolic rate (BMR) and summit metabolic rate (M_sum_ = maximum cold-induced metabolic rate) before and after acclimation (Accl). To examine mechanistic bases for metabolic variation, we measured flight muscle and heart masses and citrate synthase and β-hydroxyacyl coA-dehydrogenase activities. BMR and M_sum_ were higher for cold-acclimated than for warm-acclimated birds, and BMR was higher in winter than in summer birds. Contrary to our hypothesis of greater responses to cold Accl in winter birds, metabolic rates generally decreased over the Accl period for winter birds at all temperatures but increased at cold temperatures for summer birds. Flight muscle and heart masses were not significantly correlated with season or Accl treatment, except for supracoracoideus mass, which was lower at −10°C in winter, but flight muscle and heart masses were positively correlated with BMR and flight muscle mass was positively correlated with M_sum_. Catabolic enzyme activities were not clearly related to metabolic variation. Thus, our data suggest that predictions of the CVH may not be relevant when extended to seasonal temperature variability at the within-population scale. Indeed, these data suggest that metabolic rates are more prominently upregulated in summer than in winter in response to cold. Metabolic rates tended to decrease during Accl at all temperatures in winter, suggesting that initial metabolic rates at capture (higher in winter) influence metabolic Accl for captive birds.

## Introduction

Proper allocation of energy is a central component of life history, and adaptive reversible adjustment of metabolic rates (i.e., metabolic flexibility) to variable energy demands can prominently influence survival and fitness (Hayes and O’Connor 1999; Petit et al. 2017; Latimer et al. 2018). Evolutionary theory predicts that flexible phenotypes should evolve when the benefits of flexible responses to short-term temporal or spatial environmental variability outweigh the long-term costs of maintaining a phenotypic capacity for flexibility; therefore, a positive correlation between metabolic flexibility and environmental variability might be expected (Via and Lande 1985; DeWitt et al. 1998; Piersma and Drent 2003). Indeed, a fundamental question in ecological and evolutionary physiology is whether organisms living in highly variable environments have more flexible phenotypes. The climatic variability hypothesis (CVH) predicts that populations experiencing more variable climates should produce more flexible phenotypes (Gaston and Chown 1999; Cavieres and Sabat 2008; Naya et al. 2012), thus allowing better matching of phenotypes to current conditions.

The relationship between phenotypic flexibility and climatic variability predicted by the CVH could potentially apply to both geographic differences in climate (i.e., among-population flexibility) and intraseasonal changes in weather (i.e., within-population flexibility). For example, if one season showed more variable temperatures than another season, then within-population phenotypic flexibility might be greater during the more variable season. This requires that the capacity for phenotypic flexibility also be a flexible trait which responds to variation in short-term conditions (Boratyński et al. 2016, 2017). If so, the capacity for acclimation (Accl) might be greater in one season than in another if conditions are more variable in that season. Selection might be expected to act on within-season Accl capacity if costs of higher flexibility are greater during one season than another (e.g., maintenance of high Accl capacity in the less variable season) to optimize cost–benefit ratios. Such a scenario assumes that mechanisms underlying the capacity for flexibility can be altered over short-term temporal scales to produce the hypothesized selective advantages. Within-population, between-season, differences in flexibility could be potentially mediated by seasonal differences in stress responsiveness or regulation of gene expression (Stager et al. 2015; Boratyński et al. 2017; Cheviron and Swanson 2017), among other mechanisms.

Seasonal phenotypic flexibility allows birds inhabiting regions with cold winter climates to adjust their physiology to meet the elevated thermogenic demands of cold winters. Winter increases in both basal metabolic rate (BMR) and summit metabolic rate (M_sum_ = maximal thermogenic metabolic rate) are typically components of this flexible response (Swanson 2010), and high metabolic rates during cold winters may be positively associated with fitness in birds (Nilsson and Nilsson 2016; Petit et al. 2017; Latimer et al. 2018). Because shivering thermogenesis is the primary mechanism of heat production in birds (Marsh and Dawson 1989), increases in thermogenic capacity are mediated through adjustments in skeletal muscles, principally the pectoralis (Pec) muscle, which is the primary thermogenic organ in birds (Marsh and Dawson 1989). Adjustments in the Pec muscle resulting in enhanced organismal metabolic capacities may include increases in muscle size or cellular aerobic metabolic intensity (Marsh and Dawson 1989; Swanson 2010). Such increases in organismal metabolic capacities may also be accompanied by changes in oxygen transport, including changes in blood oxygen carrying capacity (Swanson 1990; Petit and Vézina 2014) and/or heart mass (Swanson and Vézina 2015), and/or substrate (primarily lipid) transport (Liknes et al. 2014; Zhang et al. 2015a, 2015b).

Flexible metabolic responses to climate variation could potentially involve both minimum (BMR) and maximum (M_sum_) metabolic outputs, and either, or both, could permit phenotypic matching to environmental conditions. Of these two outputs, M_sum_ is more directly related to cold tolerance capacity, as it defines the maximum capacity for thermoregulation, is positively correlated with cold tolerance both within and among species (Swanson 2001; Swanson and Liknes 2006), and is significantly associated with broad-scale patterns of geographic distribution in birds (Swanson and Garland 2009; Swanson and Bozinovic 2011; Stager et al. 2016). Winter increases in organismal metabolic capacities are common among birds in cold climates (Swanson 2010; Swanson and Vézina 2015). Seasonal patterns of metabolic flexibility are more variable for birds wintering in milder climates with lower seasonal climatic variability, and winter increases, winter decreases and seasonal stability in metabolic capacities have all been documented for tropical and subtropical birds (Wells and Schaeffer 2012; McKechnie et al. 2015; Noakes et al. 2017; Noakes and McKechnie 2020a). These variable seasonal patterns of metabolic flexibility suggest that variation in organismal metabolic rates might be correlated with seasonal climatic variation encountered by birds, and so might generally fit predictions of the CVH.

Only a few studies have examined intraspecific associations between geographic variation in metabolic flexibility and environmental heterogeneity in birds (Tieleman et al. 2003; Cavieres and Sabat 2008; Van de Ven et al. 2013a, 2013b). While some of these studies document positive associations between metabolic flexibility and environmental heterogeneity (Cavieres and Sabat 2008; Maldonado et al. 2009), others do not (Tieleman et al. 2003; van de Ven et al. 2013b; McKechnie et al. 2015; Noakes and McKechnie 2020b). To our knowledge, only three avian studies have examined relationships among M_sum_ flexibility and geographic variation in climates. van de Ven et al. (2013b) found that the magnitude of M_sum_ responses to temperature Accl were similar in two populations of southern red bishops (*Euplectes orix*) differing in seasonal temperature variability. For white-browed sparrow weavers (*Plocepasser mahali*) measured across a climatic gradient of ∼7°C in winter minimum temperature, both BMR and M_sum_ tended to be higher in winter at sites with colder climates, but no clear relationship between seasonal variation in metabolic rates and ambient temperature was evident (Noakes et al. 2017). Noakes and McKechnie (2020b) acclimated white-browed sparrow-weavers from three populations across a climatic and aridity gradient to 5, 20, and 35°C and found no variation in reaction norms for temperature Accl across populations for BMR or M_sum_, although M_sum_ was higher in the population from the coldest climate. Collectively, these studies offer some support for the hypothesis that metabolic flexibility is correlated with environmental heterogeneity, but this pattern is not universal for birds and may differ for BMR and M_sum_.

Whether among-population responses to seasonal environmental variability can be extrapolated to within-season responses is uncertain, and defining within-season capacities for flexible metabolic responses to seasonally variable climates is necessary to determine the scales at which the CVH might operate. Boratyński et al. (2016) documented greater metabolic flexibility in summer than in winter for Siberian hamsters (*Phodopus sungorus*), but this species engages in torpor and downregulates metabolism in winter associated with an energy conservation strategy. Small birds wintering in cold climates tend to show the opposite pattern of seasonal metabolic variation with an upregulation in winter. Birds can adjust metabolic rates to temperature variation over periods of days to weeks (Swanson and Olmstead 1999; Petit et al. 2013; Dubois et al. 2016), so we might expect different seasonal patterns of variation in metabolic flexibility in birds. Weekly or monthly temperature variation in winter is greater than in summer for birds in north-temperate climates, so if the relationship between temperature variability and metabolic flexibility predicted by the CVH extends to the within-population scale, the capacity for metabolic flexibility could be greater in winter than in summer for north-temperate resident birds. Additionally, temperature variation at cold temperatures well below the thermoneutral zone should evoke greater metabolic change than similar temperature variation at warm temperatures near or within the thermoneutral zone. For seasonal climates such as these, extension of the CVH to the within-population level predicts that winter individuals should exhibit greater metabolic flexibility (larger treatment differences in metabolic rates and more rapid Accl) than summer individuals due to colder and more variable winter temperatures. Alternatively, metabolic responses to temperature may remain unchanged seasonally, being determined by temperature variation over the entire year. To our knowledge, no studies have compared within-season patterns of variation in metabolic flexibility between summer and winter for birds resident in highly seasonal climates.

**Fig. 1:**
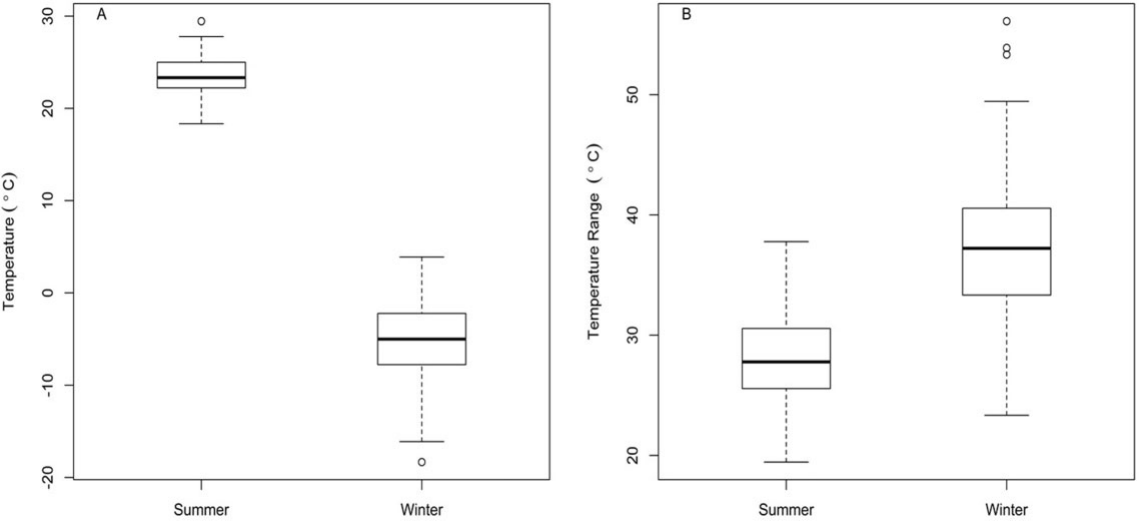
Tukey box plots of (**A**) mean (black horizontal bar) monthly temperature (daily average temperature) for Vermillion, Clay County, South Dakota in winter (December, January, and February) and summer (June, July, and August) months and (**B**) monthly temperature range (daily maximum minus daily minimum) for winter vs. summer months. We obtained temperature data from 1931 to 2011 from http://climate.sdstate.edu/coop/monthly.asp.

In this study, we tested the predictions of the within-population extension of the CVH between seasons differing in temperature variability by measuring metabolic flexibility (i.e., the magnitude of change in metabolic rates in response to temperature Accl) for both BMR and M_sum_ in summer- and winter-collected house sparrows (*Passer domesticus*) acclimated for 6 weeks to thermoneutral (24°C), cool (5°C), and cold (−10°C) temperatures. We chose house sparrows as our model species because they show prominent seasonal changes in both BMR and M_sum_ (Arens and Cooper 2005; Swanson and Liknes 2006), and these changes are supported by seasonal variation in Pec muscle and heart masses and skeletal muscle aerobic enzyme activities (Liknes and Swanson 2011a, 2011b). Moreover, they are also amenable to captive housing and show changes in metabolic rates, Pec muscle mass, and Pec aerobic enzyme activities with cold training under captive conditions (Zhang et al. 2015b, 2015c), so they serve as a good model species for this study. We hypothesized that because short-term (weekly to monthly) temperature variation is greater in winter than in summer for our study sites in South Dakota, USA (Fig. 1), the capacity for metabolic flexibility will also be greater in winter than in summer. This study allows us to test the role of within-season flexibility in organismal responses to variable environments and helps to define the scales (e.g., within vs. among populations) across which the CVH might act.

## Materials and methods

### Birds and Acclimation

We collected house sparrows by mist net near Vermillion, Clay County, South Dakota (42.8°N, 96.9°W) in mid-late May (summer birds) and early December (winter birds). We only used adult birds in these studies. At capture, we measured body mass (M_b_) to the nearest 0.1 g on an Ohaus Model LS200 (Parsippany, NJ) portable balance, length of the folded wing to the nearest 0.5 mm with a wing ruler, and tarsus length to the nearest 0.1 mm with calipers. After capture, we acclimated birds to captivity (22°C, natural photoperiod), housing them individually in 57 × 37 × 31 cm flight cages equipped with a perch, for at least 2 weeks (14–18 days). We provided food (mixed bird seed, a protein supplement consisting of a mixture of homogenized 21%-protein dog food and hard-boiled eggs, and six *Tenebrio* larvae per day per individual bird) and vitamin-enriched water (Wild Harvest Multi-Drops vitamin supplement, United Pet Group, Cincinnati, OH) during this period. After the captivity Accl period, we randomly assigned birds to one of three temperature Accl treatments (24, 5, or −10°C). We kept birds at these Accl temperatures for 6 weeks, except for the −10°C group, where we first exposed birds to 0°C for 1 week prior to reducing the temperature to −10°C for the remaining 5 weeks of the Accl treatment. We used this stepwise Accl approach for the −10°C group to acclimate birds (especially summer birds not previously exposed to cold) more gradually to this temperature. We used photoperiods for Accl for both summer and winter birds that corresponded to photoperiods (including civil twilight) at the time of the final post-Accl measurements (mid-July for summer birds and late February for winter birds) for Vermillion, South Dakota, USA. These photoperiods were 16 L:8 D for summer birds and 12 L:12 D for late winter birds. During Accl treatments, food and water, as described above, were provided ad libitum, except that we provided vitamin-enriched shaved ice for the −10°C group because water supplied in bowls froze solid at this temperature.

### Metabolic measurements

We measured BMR as a measure of maintenance metabolic costs and M_sum_ as a measure of the maximum capacity for thermogenesis. Temperatures eliciting M_sum_ in small birds are typically lower than those encountered in nature (Swanson 2010), but the utility of M_sum_ is that it is correlated with cold tolerance capacity (Swanson 2001; Swanson and Liknes 2006) and overwinter survival (Petit et al. 2017; Latimer et al. 2018) in temperate-zone birds. We measured both M_sum_ and BMR by open-circuit respirometry for each individual bird before, at the mid-point (3 weeks), and after the 6-week Accl period. We adjusted the timing of the Accl periods so that we measured metabolic rates on three birds per day, measuring M_sum_ in the morning or early afternoon and BMR the following night. Thus, each metabolic measurement period lasted a total of eight days for each Accl time point (pre, mid, and post). We used 1.9-L paint cans with the inner surface painted flat black for metabolic chambers. We measured M_sum_ using a sliding cold exposure in a 79% helium/21% oxygen (helox) atmosphere (Swanson et al. 1996), with starting temperatures at −6 to −12°C. For the sliding cold exposure, we first flushed the chamber with helox for 5 min at room temperature, then immersed the chamber into a bath (Forma Scientific Model 2095, Marietta, OH) of water-ethylene glycol capable of regulating temperature to ±0.2°C. Flow rates were maintained at 1010–1030 mL min^−1^ with a Cole-Parmer Model FM082-03ST (Vernon Hills, IL) precision rotameter calibrated with a soap bubble meter to ±1% accuracy. We dried (Drierite) and removed carbon dioxide (Ascarite) from both incurrent and excurrent air streams. We maintained the bath temperature at the initial temperature for 20 min, after which we reduced the temperature at a rate of ∼1°C every 5 min until we detected a steady decline in oxygen consumption over several minutes, which is indicative of hypothermia. We then removed the bird from the chamber and measured cloacal body temperature with a Cole-Parmer (Vernon Hills, IL) Model 8500-40 Thermocouple Thermometer by inserting a lubricated 20-gage thermocouple ∼1 cm into the cloaca to verify hypothermia. We considered birds as hypothermic if body temperature (T_b_) was < 37°C (Swanson et al. 2014), and birds were hypothermic after removal in all cases. We recorded excurrent oxygen content with an Ametek S-3A oxygen analyzer (Pittsburgh, PA) every 2 s over the measurement period using Expedata version 2.0 (Sable Systems, Henderson, NV). We calculated M_sum_ as instantaneous oxygen consumption (Bartholomew et al. 1981) using the Z transform function in Expedata version 2.0 and considered the highest 5-min mean oxygen consumption over the measurement period as M_sum_ (Swanson et al. 2012).

We kept birds in small flight cages at room temperature after M_sum_ measurement for at least 4 h, with food and water provided, prior to BMR measurement. For BMR measurement, we placed birds within the metabolic chamber at 30°C (within the thermal neutral zone for house sparrows, Nzama et al. 2010) at ∼1900 h and metabolic rates were measured overnight in air at flow rates of 280–300 mL min^−1^. We measured three birds for BMR concurrently using a 4-channel multiplexer (Sable Systems Model TR-RM4, Henderson, NV). We set the multiplexer to record oxygen consumption for 30 min sequences for each of the three birds followed by 10 min of baseline. We maintained this sequence throughout the night for at least 12 h. We recorded excurrent oxygen content with the Ametek S-3A oxygen analyzer every 2s over the measurement period using Expedata version 2.0 (Sable Systems, Henderson, NV). We calculated BMR according to steady-state equations (Lighton 2008) and considered the lowest 10-min period of oxygen consumption over the entire night as BMR for each individual bird (Zhang et al. 2015a). All metabolic rates were corrected to standard temperature and pressure dry (STPD).

### Ultrasound measurements

We measured flight muscle [Pec + supracoracoideus (Scc)] thickness to the nearest 0.01 mm on individual birds by ultrasound according to Swanson and Merkord (2013) after the captivity Accl period on the day before temperature Accl treatments began and after the 6-week Accl period, but on the day before final metabolic measurements were conducted. We conducted measurements with a Visual Sonics Vevo 770 High-Resolution Imaging system with a Model 710B scan head for ultrasound measurements of muscle thickness. We used a frequency of 25 MHz and a focal length of 15 mm for all measurements. We conducted ultrasound measurements on unanesthetized birds with a cloth bag placed over the head to calm the bird but still allow normal breathing. One person held the bird (Y.Z. or D.S.) and one person (T.A.) operated the scan head to conduct ultrasound measurements.

We wetted the plumage to expose the skin over the breast muscle and then added ultrasound recording gel to the surface of the skin above the muscle. We immersed the scan head into the recording gel until it just touched the skin surface to record measurements of the short-axis (Dietz et al. 1999) muscle thickness. To standardize the location of these measurements, we moved the scan head forward along the muscle until it just touched a metal ruler placed across the base of the furculum (Swanson and Merkord 2013). We typically took four measurements (range 3–6) of short-axis muscle thickness (Dietz et al. 1999) on each individual bird, computed the average for all measurements for each individual, excluding any outliers that differed by >20% from the mean value of the other measurements (Swanson and Merkord 2013), and used the average values in subsequent calculations.

### Tissue dissection and enzyme assays

Following the final BMR measurement, we removed sparrows from metabolic chambers and placed them in small flight cages at room temperature until dissections within 2 h after removal. We euthanized birds by cervical dislocation and excised flight muscles (Pec, Scc) and hearts quickly on the ice. We blotted tissues dry and weighed them to the nearest 0.1 mg before flash freezing in liquid nitrogen. We stored tissues at −70°C until later enzyme assays.

We measured Pec muscle and heart activities of citrate synthase (CS; E.C. E.C. 4.1.3.7), as an indicator of cellular aerobic metabolic intensity, and β-hydroxyacyl CoA dehydrogenase (HOAD, E.C. 1.1.1.35), as an indicator of β-oxidation capacity. We conducted CS and HOAD assays as previously described (Zhang et al. 2015a). After removing tissues from −70°C storage, we minced samples on ice while still partly frozen, followed by homogenization in 10–40 volumes/mass of ice-cold homogenization buffer with a Tekmar model ST-1810 Tissuemizer (Cincinnati, OH). The homogenization buffer contained 100 mM phosphate and 2 mM ethylenediaminetetraacetic acid (EDTA) at pH 7.3. Following homogenization, we used a Cole-Parmer 4710 series ultrasonic homogenizer (Chicago, IL) to sonicate tissues on ice. For sonication, we used three 10-s bursts with 30 s between successive bursts.

We conducted spectrophotometric assays for both enzymes on crude muscle homogenates at 39°C with a Beckman DU 7400 spectrophotometer (Beckman Coulter, Fullerton, CA) at 412 nm. The CS assay medium contained 100 mM triethanolamine–HCl, 2.5 mM EDTA, 0.1 mM 5.5ʹ-dithiobis-(2-nitrobenzoic acid), 0.2 mM acetyl-CoA, and 0.5 mM oxaloacetate at pH 7.5 in a final volume of 1.0 mL. The HOAD assay buffer contained 100 mM triethanolamine–HCl, 5 mM EDTA, 0.225 mM NADH_2_, and 0.1 mM acetoacetyl-CoA at pH 7.0. For both enzymes, we measured background activity for 2–3 min before adding the substrate to start the reaction for all enzymes, but background activity was negligible for both enzymes, so we did not subtract background activity from activity after addition of substrate to start the reaction. We ran each sample in duplicate and used average values for subsequent calculations and report mean mass-specific activities as µmol ⋅ min^−1^ ⋅ g wet mass^−1^.

### Statistics

Sex ratios (Male:Female) for the different Accl groups were, Winter: 24°C, 5:3; 5°C, 6:2, −10°C, 6:2; Summer: 25°C, 3:5; 5°C, 6:2; −10°C, 3:5. We used generalized linear mixed models (GLMMs) using an REML approach with M_b_, sex, season, Accl treatment (−10, 5, and 24°C), timing (pre-, mid-, and post-Accl), and season * treatment and season * treatment * timing interactions as fixed effects and individual identity as a random effect to control for repeated measures for BMR and M_sum_. We used similar GLMMs for M_b_ (without M_b_ as a covariate), and organ masses and Pec muscle and heart enzyme activities following Accl (without the timing variable as a fixed effect). For muscle and heart mass GLMMs, we used M_b_ minus the mass of the specific muscle (multiplied by two to account for both sides) or heart to avoid part-whole correlations. We conducted separate generalized linear models (GLMs) for M_b_, BMR, and M_sum_ for pre-, mid-, and post-Accl periods to assess shapes of reaction norms. Predictor variables for these GLMs included sex, season, treatment group (Trt Grp), and M_b_ (for BMR and M_sum_ only), with the season * treatment group interaction term also included. We also conducted GLMs to assess which mechanisms most affected metabolic rates. For these GLMs, we used post-Accl BMR and M_sum_ as dependent variables, with M_b_ (−2 * Pec and Scc masses, to avoid part-whole correlations), flight muscle mass [2 * (Pec + Scc mass), since we only weighed one side], heart mass and Pec and heart CS and HOAD activities as predictor variables. We also initially included interaction terms for flight muscle mass * Pec CS * Pec HOAD and heart mass * heart CS * heart HOAD, but these terms were never significant, so we removed them from subsequent analyses. We also used a backward stepwise model selection procedure with AICc to rank models to determine which mechanistic models best fit the data. To examine assumptions of the linear modeling, we visually inspected residual plots (i.e., histograms) for all dependent variables from the GLMM and GLM analyses to check that all variables were approximately normally distributed. All dependent variables were normally distributed except for Pec and heart HOAD activities, which were positively skewed, so we log_10_-transformed these values to produce normal distributions and conducted analyses on log_10_-transformed values. We used least significant difference tests as *post-hoc* tests to identify different groups when GLMMs or GLMs indicated significant differences. All GLMM and GLM analyses were conducted in IBM SPSS, version 26.0.

We used one-way analysis of variance ANOVA or Kruskal–Wallis tests, if parametric assumptions were not met, to compare among-group values within each season for ultrasound measures of flight muscle width. We used *post-hoc* Tukey tests if significant differences were detected by ANOVA. We used paired *t*-tests to compare pre-Accl and post-Accl measures of ultrasound flight muscle thickness. We calculated repeatability for ultrasound muscle width measurements as the intraclass correlation coefficient (Lessells and Boag 1987). We used the least squares regression to test the relationship between flight muscle mass and ultrasound flight muscle width. We considered *P* <0.05 as statistically significant. For statistical tests other than GLMMs, we used Sigma Stat version 3.5 (Systat Software, Inc., Point Richmond, CA).

## Results

### Seasonal temperature variation

Mean ± standard deviation (SD) winter (December–February) temperature from 1931 to 2011 for Vermillion, Clay County, South Dakota (∼42.67°N, 96.93°W) from the Vermillion cooperative weather station (http://climate.sdstate.edu/coop/monthly.asp) was −5.9 ± 3.9°C, whereas mean summer (June–August) temperature was 23.3 ± 2.0°C. The mean monthly temperature range (daily maximum to daily minimum temperatures) in winter was 37.4 ± 5.6°C, whereas that in summer was 28.3 ± 3.5°C (Fig. 1). Thus, winter temperatures were more variable and exhibited a greater difference between high and low temperatures than summer temperatures.

### M_b_ and metabolic rates

Pre-Accl M_b_ did not differ among temperature treatments in either winter or summer (Supplementary data, Table S1). The only significant effectors of M_b_ were a main effect of season and an interaction between season * Trt Grp (Table 1). Summer M_b_ was higher than winter M_b_, but this difference was driven by the −10°C Trt Grp, where the least squares mean for M_b_ was 8.6% higher in summer than in winter (*t*_122_ = 2.243, *P* < 0.001). Seasonal differences for other Trt Grps were not significant (Table 1). Post-Accl GLMs revealed that reaction norms for M_b_ differed between summer and winter, with summer birds showing a negative relationship with Accl temperature and winter birds showing no significant relationship, although the season * Trt Grp interaction term was not quite significant (Table 2).

**Table 1:** GLMM results for M_b_, BMR, and M_sum_ for house sparrows exposed to temperature Accl Trt Grps of 24°C, 5°C, and −10°C

Variable	*F* M_b_	df1	df2	*P* M_b_	*F* BMR	df1	df2	*P* BMR	*F* M_sum_	df1	df2	*P* M_sum_
M_b_	–	–	–	–	59.77	1	121	***<0.001***	17.52	1	121	***<0.001***
Sex	1.33	1	122	0.252	0.07	1	121	0.787	0.002	1	121	0.968
Season	6.48	1	122	***0.012***	54.80	1	121	***<0.001***	3.33	1	121	0.070
Trt Grp	0.71	2	122	0.495	18.45	2	121	***<0.001***	15.10	2	121	***<0.001***
Accl Timing	0.16	2	122	0.855	3.54	2	121	***0.032***	0.96	2	121	0.385
Season * Trt Grp	1.90	2	122	***0.009***	9.35	2	121	***<0.001***	5.37	2	121	***0.006***
Season * Trt Grp * Accl Timing	0.47	10	122	0.905	3.56	10	121	***<0.001***	2.32	10	121	***0.015***

Accl timing effects were from repeated measures before Accl and at 3 and 6 weeks of Accl to the temperature treatments. Significant *P*-values are shown in bold italics.

**Table 2:** GLMM results for pre-, mid-, and post-Accl treatments for M_b_, BMR, and M_sum_ for house sparrows exposed to temperature Accl Trt Grps of 24°C, 5°C, and −10°C

Variable	*F* M_b_ Pre	df1	df2	*P*-value	*F* M_b_ Mid	df1	df2	*P*-value	*F* M_b_ Post	df1	df2	*P*-value
Sex	0.009	1	40	0.925	0.908	1	40	0.346	1.304	1	40	0.260
Season	2.229	1	40	0.143	3.050	1	40	0.088	1.297	1	40	0.262
Trt Grp	0.172	2	40	0.842	0.691	2	40	0.507	0.758	2	40	0.475
Season * Trt Grp	0.179	2	40	0.837	2.718	2	40	0.078	3.129	2	40	0.055
	***F* BMR Pre**				***F* BMR Mid**				***F* BMR Post**			
M_b_	23.132	1	39	***<0.001***	35.087	1	39	***<0.001***	8.520	1	39	***0.006***
Sex	0.000	1	39	0.987	0.640	1	39	0.428	0.068	1	39	0.796
Season	79.312	1	39	***<0.001***	8.569	1	39	***0.006***	2.380	1	39	0.131
Trt Grp	4.011	2	39	***0.026***	10.423	2	39	***<0.001***	5.072	2	39	***0.011***
Season * Trt Grp	6.863	2	39	***0.003***	3.244	2	39	***0.050***	1.572	2	39	0.220
	***F* M_sum_ Pre**				***F* M_sum_ Mid**				***F* M_sum_ Post**			
M_b_	5.934	1	39	***0.020***	7.462	1	39	***0.009***	5.289	1	39	***0.027***
Sex	0.456	1	39	0.503	0.000	1	39	0.993	0.145	1	39	0.706
Season	6.465	1	39	***0.015***	12.115	1	39	***0.001***	0.642	1	39	0.428
Trt Grp	13.397	2	39	***<0.001***	5.275	2	39	***0.009***	2.695	2	39	0.080
Season * Trt Grp	5.323	2	39	***0.009***	2.309	2	39	0.113	0.812	2	39	0.451

Significant *P*-values are shown in bold italics.

After 2 weeks of captivity at room temperature (22°C), mean BMR for pre-Accl sparrows in winter significantly exceeded that in summer sparrows by 19.7% (Supplementary data, Table S1). Mean M_sum_ for pre-Accl sparrows in winter was only 4.3% higher in winter than in summer, a nonsignificant difference (Supplementary data, Table S1).

**Fig. 2:**
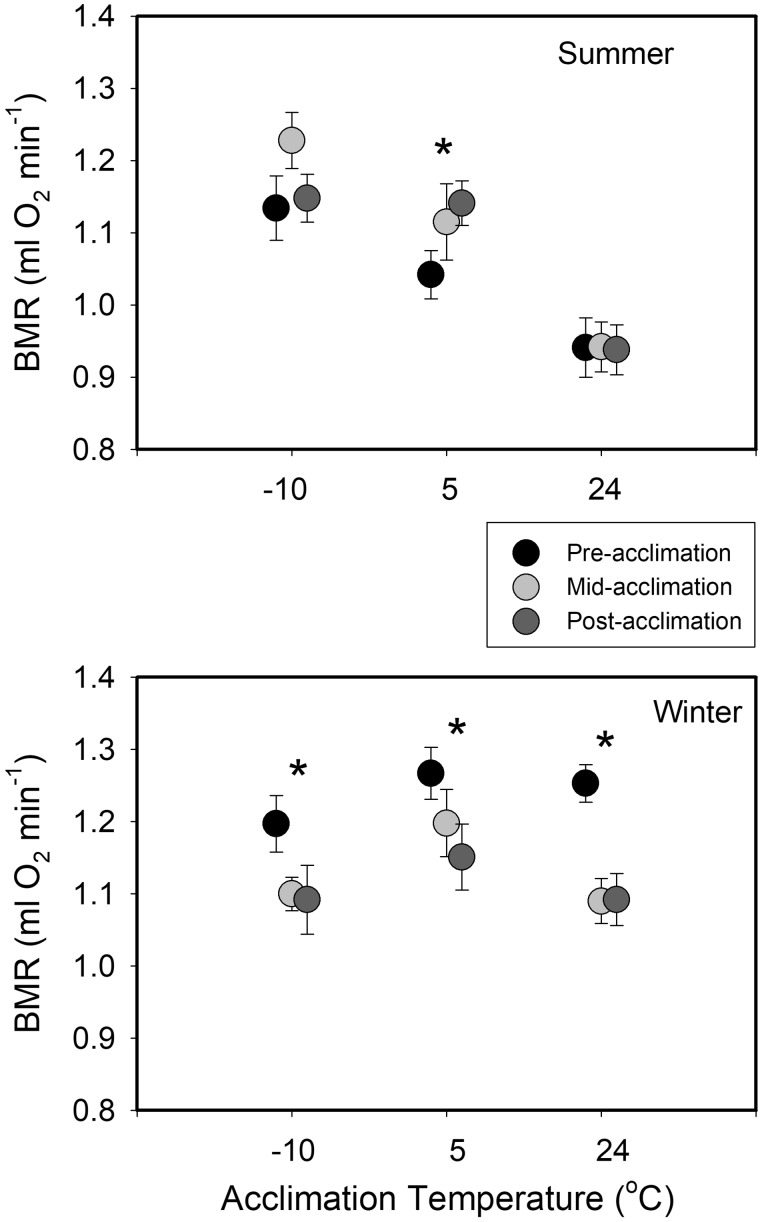
Temporal trends in mean ± SE BMR as a function of Accl temperature in winter and summer house sparrows from South Dakota, USA. Pre-Accl measurements occurred the day prior to Accl. Mid-Accl measurements occurred after 3 weeks and post-Accl measurements occurred after the full 6-week Accl period. Significant differences among Accl periods from GLMM analyses are denoted by an asterisk.

Sex was not a significant predictor of BMR, but all other main effects and interaction terms showed significant effects on BMR (Table 1). As expected, M_b_ was significantly positively related to BMR. BMR was higher during winter than during summer, with least squares mean BMR 10.9% greater in winter than in summer (Fig. 2). In addition, BMR for warm-acclimated sparrows (24°C) was lower than in other Accl groups, with least squares mean BMR at 24°C 8.3% lower than for 5°C and −10°C Trt Grps (Fig. 2). The significant season * Trt Grp interaction (Table 1) indicated that the effect of season on BMR was not significant under the coldest (−10°C) Accl treatment, but was significant, with higher values in winter than in summer for both 5°C and 24°C treatments. The significant season * Trt Grp * Accl timing interaction indicated that the effect of season on the BMR response to the Accl (temperature) treatments was also influenced by the timing of measurements during the Accl period. Seasonal differences were greatest for pre-Accl measurements, for which winter BMR measurements averaged 19.7% higher than summer BMR (Fig. 2). Patterns of variation with Accl timing also differed among seasons, with winter birds showing significant reductions from pre-Accl BMR at all temperatures for both mid- and post-Accl measurements (all *P* < 0.05), whereas summer birds showed a general pattern of increasing BMR at cold temperatures over the Accl period, but this was significant only for the pre- vs. post-Accl measurement at 5°C (Fig. 2). Reaction norms for BMR with Accl temperature showed the same seasonal trends as for M_b_, with a negative relationship in summer (although the two cold Trt Grps did not differ significantly from each other) and no significant relationship in winter (Table 2). Interestingly, this same trend was also evident for pre-Accl BMR in summer, but BMR values for the two cold groups in summer converged after the 6-week Accl period (Fig. 2). Season was a significant predictor for pre- and mid-Accl BMR, but not for post-Accl BMR (Table 2), suggesting that Accl to cold resulted in seasonal convergence of BMR, which was higher in winter for pre- and mid-Accl birds (Table 1).

**Fig. 3:**
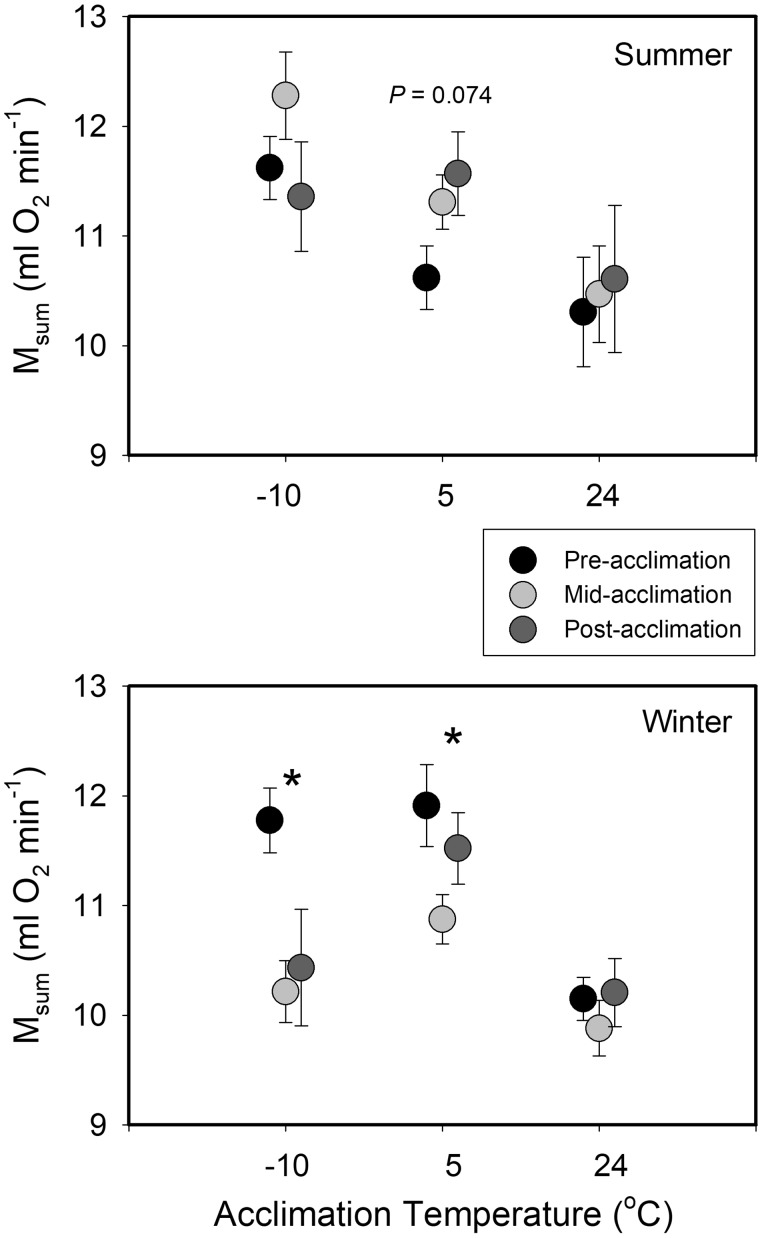
Temporal trends in mean ± SE M_sum_ as a function of Accl temperature in winter and summer house sparrows from South Dakota, USA. Pre-Accl measurements occurred the day prior to Accl. Mid-Accl measurements occurred after 3 weeks and post-Accl measurements occurred after the full 6-week Accl period. Significant differences among Accl periods from GLMM analyses are denoted by an asterisk and nonsignificant trends are identified by *P*-values over the Accl temperature group.

For the GLMM for M_sum_, main effects of sex, season, and Accl timing were not significant, although the season effect approached significance, with the overall least squares mean M_sum_ being 14.5% higher than in summer (Table 1). All other main effects and interaction terms showed significant effects on M_sum_ (Table 1). M_b_ was significantly positively correlated with M_sum_ (Table 1). The main effect of Trt Grp was also significant, with cold treatments (combined) having a 15.4% higher least squares mean M_sum_ than the 24°C Trt Grp (Table 1). The season * treatment interaction indicated that the higher M_sum_ during winter was driven largely by the 5°C Trt Grp, where least squares mean M_sum_ was 31.0% higher in winter than in summer (Fig. 3). In addition, the significant season * Trt Grp * Accl timing interaction indicated that summer birds tended to increase M_sum_ over the Accl period at cold temperatures, although the only comparison that approached significance was pre- vs. post-Accl at 5°C. In contrast, winter birds tended to decrease M_sum_ over the Accl period at cold, but not warm temperatures (Fig. 3), with pre-Accl least squares mean M_sum_ values significantly higher than mid-Accl values at 5°C, and higher than both mid- and post-Accl values at −10°C. Neither summer nor winter post-Accl M_sum_ showed a significant relationship with temperature, although summer birds showed a nonsignificant trend toward a negative relationship (Table 2), suggesting flat M_sum_ vs. Accl temperature reaction norms at both seasons.

### Ultrasound flight muscle measurements and flight muscle and heart masses

**Fig. 4:**
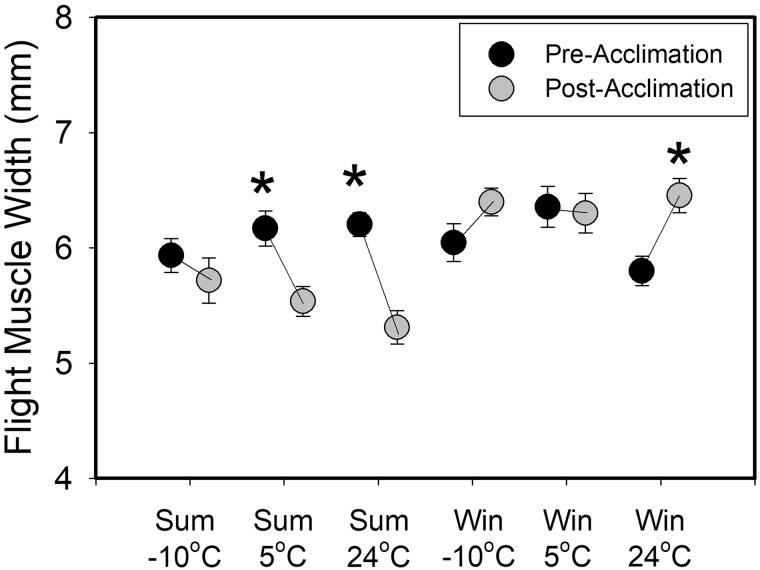
Temporal trends in mean ± SE short-axis flight muscle width measured by ultrasound as a function of Accl temperature in winter (Win) and summer (Sum) house sparrows from South Dakota, USA. Pre-Accl measurements occurred the day prior to Accl and post-Accl measurements occurred after the full 6-week Accl period. Significant differences between pre- and post-Accl periods are denoted by an asterisk.

Repeatabilities for ultrasound measurements were 0.678 for pre-Accl measurements and 0.868 for post Accl measurements. Post-Accl muscle width measured by ultrasound was significantly positively correlated with flight muscle mass (Pec + Scc) after dissection (*r* = 0.316, *F*_1,45_  = 5.007, *P* = 0.030), but variation in muscle width only explains ∼10% of flight muscle mass. Flight muscle width measured by ultrasound did not differ among Accl groups for pre-Accl or post-Accl periods at either season, although the pre-Accl flight muscle width for winter birds tended to be smaller for the 24°C Accl treatment (4.1–8.7%). Within-group differences between pre- and post- Accl periods, however, were apparent in both summer and winter. For summer sparrows, flight muscle width declined significantly from pre- to post-Accl periods for both 24°C and 5°C Accl treatments, but not for the −10°C Accl treatment (Fig. 4). In contrast, flight muscle width in winter sparrows increased significantly for the 24°C Accl treatment, but not for the other two groups (Fig. 4).

**Fig. 5:**
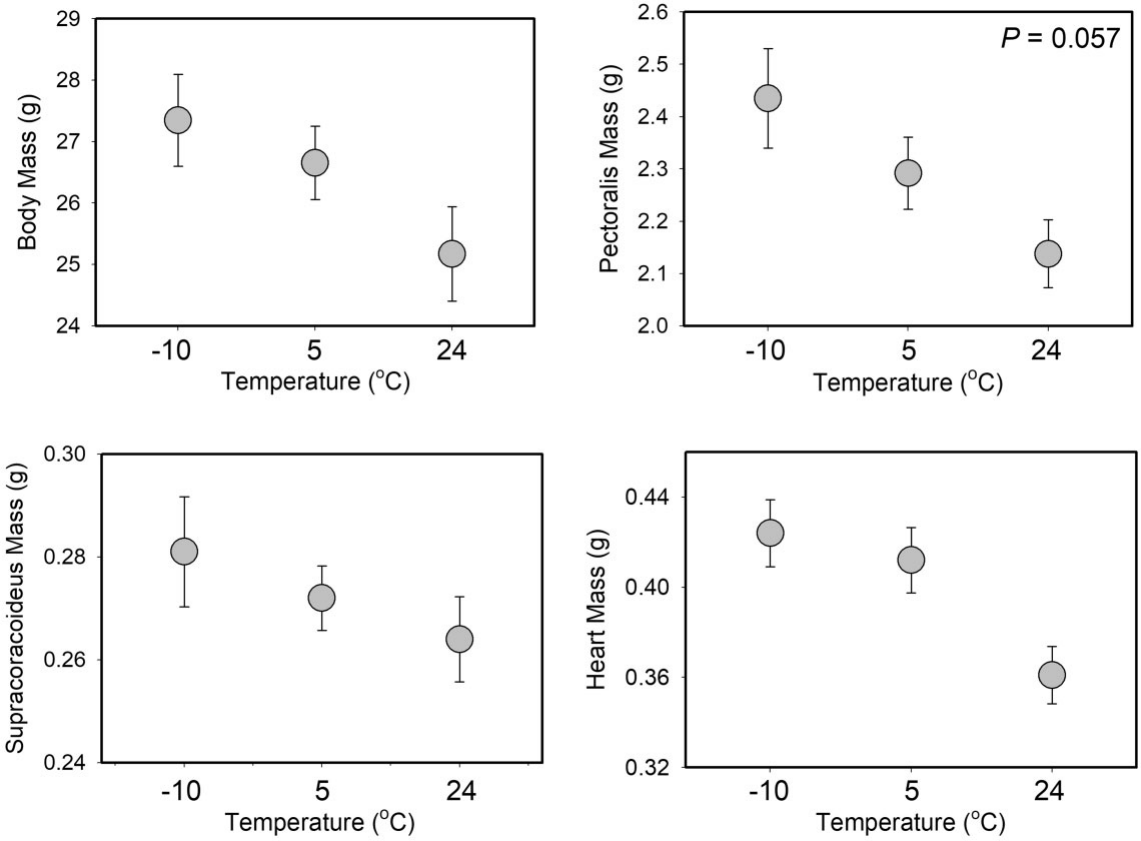
Mean ± SE body, flight muscle (one side only) and heart masses after 6 weeks of Accl as a function of Accl treatment for summer house sparrows from South Dakota, USA. No significant differences among Accl treatments were detected in summer, although the difference in Pec mass between −10°C and 24°C treatments was close to significant (*P* = 0.057).

**Fig. 6:**
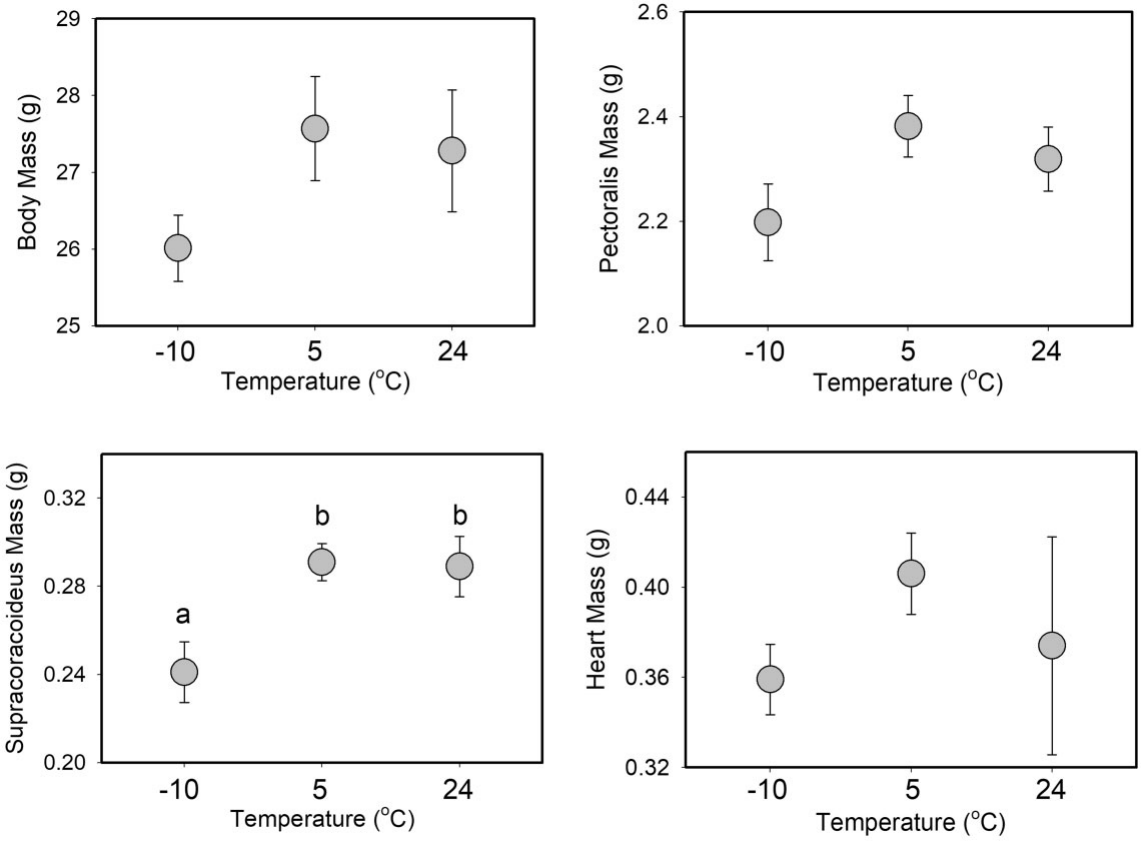
Mean ± SE body, flight muscle (one side only) and heart masses after 6 weeks of Accl as a function of Accl treatment for winter house sparrows from South Dakota, USA. Significant differences among groups from GLMM analyses are denoted by differing letters over the bars.

The only significant predictor of Pec muscle mass (Supplementary data, Table S2 for means ± standard error, SE) was M_b_, which was positively associated with Pec mass (Table 3). The season * Accl treatment interaction, however, was marginally non-significant, with the −10°C group having 7.2% larger Pec mass than the 24°C group in summer (Figs. 5, 6 and Table 3). M_b_ and the season * Accl treatment interaction were both significant effectors of Scc mass (Table 3). M_b_ was positively correlated with Scc mass. The driver of the season * Accl treatment interaction was the 24 and 5°C groups having higher Scc mass than the −10°C group in winter (17.1 and 15.4%, respectively), but not in summer (Figs. 5 and 6).

**Table 3:** GLMM results for flight muscle (Pec and Scc) and heart (Hrt) masses for house sparrows exposed to temperature Accl Trt Grps of 24°C, 5°C, and −10°C

Variable	*F* Pec	df1	df2	*P* Pec	*F* Scc	df1	df2	*P* Scc	*F* Hrt	df1	df2	*P* Hrt
M_b_	29.16	1	39	***<0.001***	13.61	1	39	***0.001***	11.77	1	39	***0.001***
Sex	0.14	1	39	0.707	1.91	1	39	0.175	5.02	1	39	***0.031***
Season	0.74	1	39	0.396	0.08	1	39	0.779	3.57	1	39	0.066
Trt Grp	0.23	2	39	0.796	2.94	2	39	0.065	1.72	2	39	0.192
Season * Trt Grp	2.82	2	39	0.072	3.33	2	39	***0.046***	1.58	2	39	0.218

M_b_ values used in the analyses were M_b_ minus muscle or heart masses to avoid part-whole correlations. Significant *P*-values are shown in bold italics.

Heart mass (Supplementary data, Table S2 for means ± SE) was significantly influenced by M_b_ and sex (Table 3), but not by other predictor variables (Figs. 5 and 6, Table 3). M_b_ was positively correlated with heart mass and heart mass in males exceeded that in females by 7.0%.

### Catabolic enzyme activities

M_b_ was not a significant effector of enzyme activity for any of the tissues or enzymes, so we removed it and ran GLMM analyses without M_b_.

**Fig. 7:**
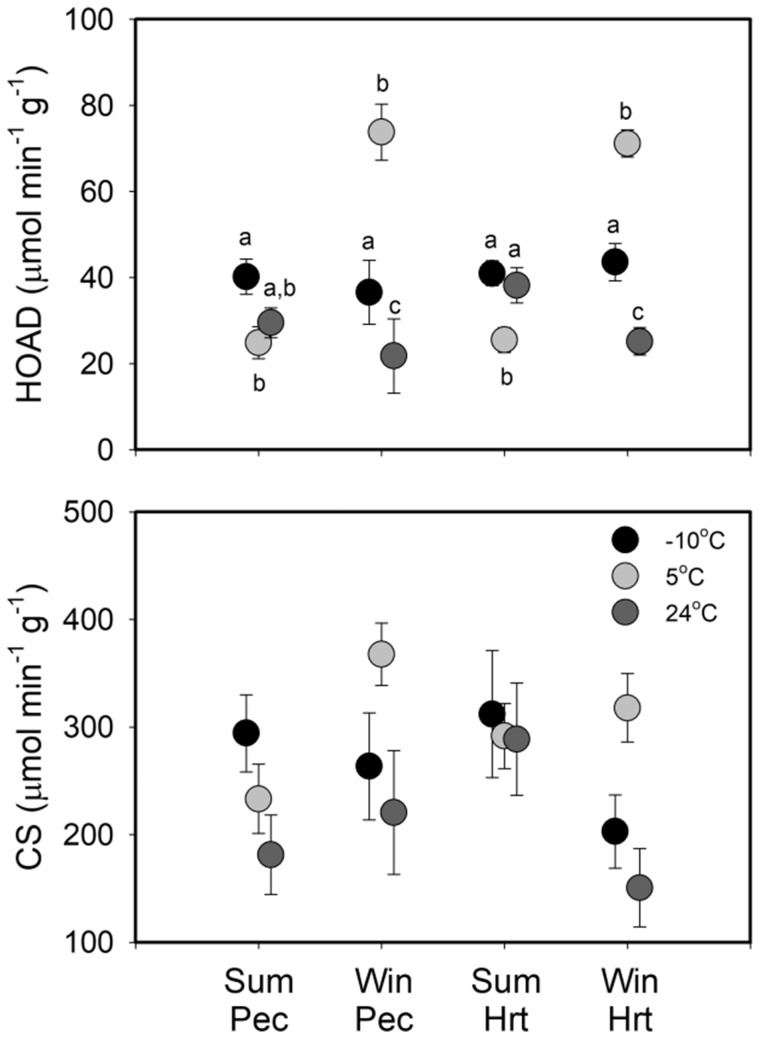
Mean ± SE Pec and heart (Hrt) activities for CS and HOAD after 6 weeks of Accl as a function of Accl treatment for winter (Win) and summer (Sum) house sparrows from South Dakota, USA. Significant differences among groups from GLMM analyses are denoted by differing letters over the bars.

Pec CS activity (Supplementary data, Table S2 for means ± SE for enzyme activities) was not significantly influenced by any predictor variables (Table 4). Pec HOAD activity showed more variation, with Accl treatment and the season * treatment interaction significantly affecting enzyme activity (Table 4). Cold-acclimated sparrows showed higher Pec HOAD activity than 24°C sparrows, with the combined mean for cold-acclimated birds 44.2% greater than for 24°C-acclimated birds. Accl treatments affected Pec HOAD activity differently in summer and winter, with mean activity for the −10°C group exceeding that in the 5°C group by 61.4% in summer. For winter birds, the 5°C group showed the highest Pec HOAD activity, exceeding that in the −10°C group by 202% and in the 24°C group by 339% (Fig. 7). Pec HOAD activity in winter birds was also significantly greater in the −10°C group than in the 24°C group (Fig. 7).

**Table 4:** GLMM results for Pec muscle and heart (Hrt) CS, and HOAD activities for house sparrows exposed to temperature Accl Trt Grps of 24°C, 5°C, and −10°C

Variable	*F* Pec CS	df1	df2	*P* Pec CS	*F* Pec HD	df1	df2	*P* Pec HD	*F* Hrt CS	df1	df2	*P* Hrt CS	*F* Hrt HD	df1	df2	*P* Hrt HD
Sex	0.07	1	36	0.787	0.53	1	39	0.470	0.006	1	36	0.937	0.05	1	39	0.832
Season	1.92	1	36	0.175	1.43	1	39	0.239	0.16	1	36	0.689	7.04	1	39	***0.011***
Trt Grp	2.80	2	36	0.074	22.29	2	39	***<0.001***	2.21	2	36	0.124	8.26	2	39	***0.001***
Season * Trt Grp	2.00	2	36	0.150	23.20	2	39	***<0.001***	2.12	2	36	0.135	28.95	2	39	***<0.001***

Significant *P*-values are shown in bold italics.

No variables were significantly associated with CS activity in house sparrow heart (Table 4). Similar to Pec muscle, however, heart HOAD activity showed more variation, with season, Accl treatment, and the season * treatment interaction all significantly affecting activity (Table 4). Mean heart HOAD activity in winter sparrows exceeded that in summer sparrows by 33.7%. Cold-acclimated sparrows had higher heart HOAD activity than 24°C-acclimated sparrows, with the combined least squares mean for 5°C and −10°C groups exceeding that for the 24 °C—acclimated group by 43.0%. Seasonal differences in Accl treatments were also evident (Fig. 7). The combined mean for 24°C and −10°C groups exceeded that for the 5°C group by 55.3% in summer. In contrast, the 5°C group showed the highest Pec HOAD activity in winter, exceeding that in the −10°C group by 63.3% and in the 24°C group by 283% (Fig. 7). Heart HOAD activity in winter birds was also significantly greater in the −10°C group than in the 24°C group (Fig. 7).

### Mechanistic correlates of metabolic rates

The top-ranked mechanistic model for BMR included only flight muscle and heart masses and no other models had ΔAICc within 4. For this model, both heart (*P* = 0.028) and flight muscle (*P* = 0.045) masses were significantly positively related to BMR. For M_sum_, the top-ranked mechanistic model included M_b_, flight muscle mass and heart CS activity, with no other models having a ΔAICc within 4. Of these variables, only flight muscle mass was a significant predictor, and was positively correlated (*P* = 0.031) with M_sum_.

## Discussion

The CVH predicts that greater variability in environmental conditions will produce greater physiological flexibility in populations exposed to such heterogeneous environments (Gaston and Chown 1999; Cavieres and Sabat 2008; Naya et al. 2012). If this predicted relationship is extended to the within-population between-seasons level, given the greater variation in temperatures during winter than during summer at temperate latitudes, such as those in the South Dakota study sites in this study, greater physiological flexibility in winter than in summer might be expected. Our data from house sparrows in this study were not consistent with this prediction, as organismal metabolic rates did not show greater responses to cold Accl in winter than in summer. Indeed, metabolic rates increased in response to cold exposure in summer, but the opposite pattern occurred in winter. These data suggest that extrapolation of the CVH to the within-population level may not be appropriate.

House sparrows in our study showed no strong seasonal trends in metabolic flexibility for either BMR or M_sum_, but upregulation of metabolic rates in response to cold temperatures tended to be stronger in summer, when metabolic rates were at their seasonal low (Arens and Cooper 2005; Swanson and Liknes 2006; Oboikovitz 2018). As far as we are aware, no studies have examined the extension of the CVH to within-population, between-season, metabolic flexibility in birds. One study, however, has addressed this question in mammals. Boratyński et al. (2016) studied Siberian hamsters (*P. sungorus*), a species that downregulates BMR in winter for energy conservation, acclimated to simulated summer and winter conditions. Hamsters in this study were first exposed to one temperature of a series (1, 20, or 28°C) for 3 weeks and then exposed to a second temperature in this series for an additional 3 weeks, with BMR measured at both 3 and 6 weeks. Metabolic flexibility was lower in winter-acclimated than in summer-acclimated hamsters and was associated with a reduction in M_b_ in winter-acclimated animals consistent with an energy conservation strategy (Boratyński et al. 2016). How these results relate to the within-population, between-season predictions of the CVH is uncertain because even though winter hamsters are active daily and forage on the surface, they also occupy borrows and use daily heterothermy and huddling (Jefimow et al. 2011), so whether the temperatures to which they are exposed are more variable in winter than in summer is uncertain.

The opposing seasonal directions of metabolic rate changes in response to cold Accl in the present study may be a function of the initial starting point for metabolic rates in naturally acclimatized birds. Resident birds wintering in cold climates typically have higher BMR and M_sum_ than summer birds (McKechnie 2008; Swanson 2010). Winter house sparrows from North America have an average M_sum_ 32% higher than summer sparrows from the same locations (South Dakota: Swanson and Liknes 2006; Swanson and Merkord 2013; Oboikovitz 2018; Ontario: Hart 1962; Wisconsin: Arens and Cooper 2005). BMR in house sparrows from Wisconsin was 62% higher in winter than in summer (Arens and Cooper 2005), although South Dakota populations only showed a non-significant 11% increase in winter (Oboikovitz 2018). South African house sparrows, from a mild temperate climate, showed the greatest seasonal variation in BMR, with winter BMR 2.1-fold greater than summer (Nzama et al. 2010). In the present study, even after 2 weeks of Accl to room temperature (22°C), mean BMR in winter exceeded that in summer by almost 20%, although M_sum_ in winter was only 4% higher in winter than in summer, a nonsignificant difference. Thus, the seasonal set-points for metabolic rates under natural conditions appear to influence seasonal patterns of responses to temperature Accl. Winter birds in this study downregulated metabolic rates from initially high values during captive conditions at all Accl temperatures. Summer birds, in contrast, generally upregulated metabolic rates from low initial values when exposed to cold Accl temperatures.

The opposing seasonal trends in Accl responses to temperature were not clearly associated with differences in muscle masses among Trt Grps, as the only significant difference in winter birds was a reduction in Scc mass at −10°C relative to other groups. Limits to isometric shivering capacity in birds are potentially mediated by the smaller of the antagonistic flight muscles, the Scc (Marsh and Dawson 1989). Subsequent research, however, suggests that Scc mass does not appear to limit either shivering or flight metabolic capacities in birds (Liknes and Swanson 2011a, 2011b; Swanson et al. 2013; Jehl et al. 2015). Post-Accl M_sum_ did not differ significantly among winter Trt Grps, so the downregulation of Scc mass for winter birds at −10°C is likely not a driver of metabolic Accl in this study. In addition, the only significant change in ultrasound flight muscle thickness with temperature Accl for winter birds was an increase at 24°C. M_sum_ was stable after Accl at 24°C in winter, perhaps because the 2-week captivity Accl period provided a “head-start” to Accl at this temperature, so the thicker muscle after Accl in this group did not contribute to an increase in M_sum_. In addition, BMR was reduced after Accl in this group, so the thicker flight muscles were not related to changes in either BMR or M_sum_. A similar lack of correlation between flight or pectoral muscle size and metabolic capacities have been documented for other passerines (Barceló et al. 2017; Milbergue et al. 2018), despite the generally positive relationship between flight muscle size and metabolic capacity in birds (Swanson 2010).

Pec and heart masses typically increase in winter in temperate-zone birds (Swanson 2010) and positive correlations of Pec or heart masses with maximal metabolic capacities within individual birds are also common (Chappell et al. 1999; Swanson et al. 2013; Petit and Vézina 2014; Petit et al. 2014), but neither were significantly influenced by cold Accl during either season in this study. Flight muscle and heart masses, however, were significant predictors of BMR in this study, and both were positively correlated with BMR. These data are consistent with numerous other bird studies (e.g., Chappell et al. 1999; Liknes and Swanson 2011a, 2011b) and highlight that the large size of the Pec muscle, even when not actively contracting, carries substantial maintenance costs. M_sum_ was also positively correlated with flight muscle mass in this study, which has also been documented for numerous other bird studies (Swanson et al. 2013; Petit and Vézina 2014), although several recent studies suggest that the positive relationship between flight or Pec muscle masses and M_sum_ is not inviolate for birds (Swanson et al. 2014; Barceló et al. 2017; Milbergue et al. 2018; Noakes et al. 2020).

The absence of Pec muscle and heart mass variation in response to cold in this study also does not support the hypothesis of enhanced capacity for metabolic flexibility in the cold for winter birds. Such a result is consistent with results for white-throated sparrows (*Zonotrichia albicollis*, Barceló et al. 2017) and black-capped chickadees (Milbergue et al. 2018), which demonstrated increases in thermogenic capacity without corresponding increases in flight muscle masses. Interestingly, however, ultrasound muscle thickness decreased between pre- and post-Accl summer birds at 24 and 5°C, but not at −10°C, suggesting that preservation of Pec muscle size at −10°C may have contributed to maintenance of shivering capacity in cold-acclimated birds. The ultrasound results must be interpreted with caution, however, as ultrasound muscle width only explained ∼10% of the variation in flight muscle mass in this study. For summer birds, trends among Trt Grps for flight muscle mass and ultrasound measurements of muscle width were identical, with the lowest values for the 24°C group, intermediate values for the 5°C group, and highest values for the −10°C group, although differences among groups by one-way ANOVA were significant only for flight muscle mass (*P* = 0.046 for mass, *P* = 0.208 for width). Percent differences among groups ranged from 3.3% to 7.7% for muscle width and 6.0–13.1% for muscle mass. Thus, for summer birds the ultrasound measurements seemed to work well to describe flight muscle mass. For winter birds, the among-group differences were smaller, and the patterns of variation were not the same between mass and ultrasound measurements. For mass, Trt Grps varied as 5°C > 24°C > −10°C; for ultrasound Trt Grps varied as 24°C > −10°C > 5°C. Percent differences among groups ranged from 0.9% to 2.4% for muscle width and 2.5–9.5% for muscle mass, and no differences were even close to significance. Thus, ultrasound measurements tracked differences in summer birds well, but performed less well in winter birds, where among-group differences were lower. Discrepancies between measurements of ultrasound flight muscle width and Pec muscle mass in this study were of similar magnitude to the Swanson and Merkord (2013) study of house sparrows. Similar results for ultrasound measurements of muscle width not precisely tracking changes in flight muscle mass have also been reported for other birds (Royer-Boutin et al. 2015; Noakes et al. 2020) and suggest that fairly large changes in muscle mass are needed for ultrasound measurements to detect differences.

Trends in aerobic enzyme capacities in Pec and heart also were not clearly related to metabolic variation in this study. Few differences in Pec or heart CS activities occurred among temperature Accl treatments. HOAD activity showed more variation with temperature Accl, but not in a consistent direction. Pec and heart HOAD activity was generally higher in winter at 5°C than other temperature treatments, but this higher activity was not associated with upregulation of either BMR or M_sum_. HOAD activity in summer was generally lower at 5°C than for other Accl treatments for both Pec and heart, but again, these differences were not positively associated with differences among Accl groups in BMR and M_sum_. Thus, the overall pattern was for little association between aerobic enzyme activities and metabolic capacities for sparrows in this study. This is consistent with variable correlations of Pec and heart enzyme activities with metabolic capacities in seasonally acclimatized or cold-acclimated small birds generally (Marsh and Dawson 1982; O’Connor 1995; Liknes and Swanson 2011b; Zheng et al. 2013, 2014; Peña-Villalobos et al. 2014; Zhang et al. 2015a; Hu et al. 2017; Wang et al. 2019).

## Conclusions

Overall, cold Accl of house sparrows in summer and winter resulted in opposing trends in metabolic rates, with increases in summer and decreases in winter, despite the higher variability in ambient temperatures in winter relative to summer at our study sites. Thus, these data are not consistent with predictions of the CVH (Gaston and Chown 1999; Cavieres and Sabat 2008; Naya et al. 2012) when extrapolated to the within-population between-season level, suggesting that the CVH may not apply to this scale in birds. Moreover, metabolic variation with cold Accl was not consistently related to variation in skeletal muscle or heart masses or aerobic enzyme activities among Trt Grps at either season. The greater upregulation of summer metabolic rates in response to cold is consistent with the seasonal pattern of flexibility of temperature–metabolism reaction norms in Siberian hamsters (Boratyński et al. 2016). This similarity occurred despite hamsters showing metabolic downregulation in winter (Boratyński et al. 2016), whereas temperate birds generally upregulate metabolic capacities in winter (Swanson 2010; Swanson and Vézina 2015). Instead, the starting points for metabolic rates from wild birds at collection (higher in winter than in summer), appear to influence temperature Accl responses for captive birds in this study. Captivity effects on metabolic rates (e.g., Swanson and King 2013) might also influence the seasonal patterns of metabolism–temperature relationships. Future research exposing birds to colder and fluctuating (Vézina et al. 2006) temperatures at different seasons or after different periods of captivity might help tease apart the interacting effects of captivity, season, and temperature on avian metabolism–temperature relationships.

## Supplementary Material

obaa039_Supplementary_Data
