# Supplementary material for: Metabolic Flexibility in Response to Within-Season Temperature Variability in House Sparrows
Source: Integr Org Biol. 2020 Nov 5;2(1):obaa039. doi: 10.1093/iob/obaa039 (PMC7810579; doi:10.1093/iob/obaa039)
Supplement: obaa039_Supplementary_Data [file obaa039_supplementary_data.zip › Table S1.docx]

| **Trait/Season/Treatment** | **n** | **Pre-acclimation** | **Mid-acclimation** | **Post-acclimation** |
| --- | --- | --- | --- | --- |
| *M_b_ - Summer* |  |  |  |  |
| 24°C | 8 | 27.6 ± 0.9 | 26.6 ± 0.8 | 26.2 ± 0.8 |
| 5°C | 8 | 27.5 ± 0.6 | 27.9 ± 0.7 | 27.8 ± 0.5 |
| -10°C | 7 | 27.6 ± 0.8^a^ | 28.9 ± 0.8^a^ | 28.4 ± 0.7^a^ |
| *M_b_ - Winter* |  |  |  |  |
| 24°C | 8 | 27.0 ± 0.5 | 27.1 ± 0.7 | 27.3 ± 0.8 |
| 5°C | 8 | 26.9 ± 0.7 | 27.5 ± 0.7 | 27.6 ± 0.7 |
| -10°C | 8 | 25.3 ± 0.5 | 26.2 ± 0.4 | 26.0 ± 0.4 |
| *BMR - Summer* |  |  |  |  |
| 24°C | 8 | 0.941 ± 0.045^a,b^ | 0.942 ± 0.034^b^ | 0.938 ± 0.035^b^ |
| 5°C | 8 | 1.042 ± 0.034^a,c^ | 1.115 ± 0.053 | 1.141 ± 0.031 |
| -10°C | 7 | 1.134 ± 0.045 | 1.228 ± 0.039 | 1.148 ± 0.033 |
| *BMR - Winter* |  |  |  |  |
| 24°C | 8 | 1.253 ± 0.026^c^ | 1.090 ± 0.031 | 1.092 ± 0.036 |
| 5°C | 8 | 1.267 ± 0.036 | 1.198 ± 0.046 | 1.151 ± 0.046 |
| -10°C | 8 | 1.197 ± 0.039 | 1.100 ± 0.023 | 1.092 ± 0.048 |
| *M_sum_ - Summer* |  |  |  |  |
| 24°C | 8 | 10.306 ± 0.498 | 10.468 ± 0.441 | 10.607 ± 0.670 |
| 5°C | 8 | 10.619 ± 0.289 | 11.308 ± 0.248 | 11.567 ± 0.382 |
| -10°C | 7 | 11.619 ± 0.288 | 12.279 ± 0.398 | 11.357 ± 0.499 |
| *M_sum_ - Winter* |  |  |  |  |
| 24°C | 8 | 10.151 ± 0.196 | 9.882 ± 0.254 | 10.208 ± 0.310 |
| 5°C | 8 | 11.911 ± 0.374^a^ | 10.876 ± 0.224^c^ | 11.521 ± 0.325 |
| -10°C | 8 | 11.777 ± 0.295^c^ | 10.216 ± 0.281 | 10.434 ± 0.532 |
| *Pectoralis Width - Summer* |  |  |  |  |
| 24°C | 8 | 6.202 ± 0.102 | ----- | 5.309 ± 0.144^b^ |
| 5°C | 8 | 6.167 ± 0.154 | ----- | 5.536 ± 0.130^b^ |
| -10°C | 7 | 5.934 ± 0.146 | ----- | 5.717 ± 0.196 |
| *Pectoralis Width - Winter* |  |  | ----- |  |
| 24°C | 8 | 5.800 ± 0.128 | ----- | 6.454 ± 0.148^b^ |
| 5°C | 8 | 6.356 ± 0.178 | ----- | 6.300 ± 0.173 |
| -10°C | 8 | 6.045 ± 0.162 | ----- | 6.397 ± 0.120 |

**Table S1**. Mean (± SE) values for body mass (M_b_, g), basal (BMR) and summit (M_sum_) metabolic rates (ml O_2_ min^-1^), and ultrasound pectoralis muscle width (mm) for house sparrows acclimated in summer and winter to 24, 5, and -10°C for six weeks. Pre-acclimation refers to measurements before acclimation treatments began, Mid-acclimation to measurements after three weeks, and Post-acclimation to measurements after six weeks. Superscripts refer to significant differences between seasons within treatment groups (a), between treatment groups within a season (b), or with acclimation timing within treatment groups (c).
