# Supplementary material for: Metabolic Flexibility in Response to Within-Season Temperature Variability in House Sparrows
Source: Integr Org Biol. 2020 Nov 5;2(1):obaa039. doi: 10.1093/iob/obaa039 (PMC7810579; doi:10.1093/iob/obaa039)
Supplement: obaa039_Supplementary_Data [file obaa039_supplementary_data.zip › Table S2.docx]

| **Trait/Season** | **24°C** | **5°C** | **-10°C** |
| --- | --- | --- | --- |
| *Pectoralis Mass* |  |  |  |
| Summer | 2.138 ± 0.065 (8) | 2.292 ± 0.069 (8) | 2.435 ± 0.095 (7) |
| Winter | 2.319 ± 0.061 (8) | 2.382 ± 0.059 (8) | 2.198 ± 0.073 (8) |
| *Supracoracoideus Mass* |  |  |  |
| Summer | 0.264 ± 0.008 (8) | 0.272 ± 0.006 (8) | 0.281 ± 0.011 (7) |
| Winter | 0.289 ± 0.014 (8) | 0.291 ± 0.008 (8) | 0.241 ± 0.014^a^ (8) |
| *Heart Mass* |  |  |  |
| Summer | 0.361 ± 0.013 (8) | 0.419 ± 0.015 (8) | 0.424 ± 0.015 (7) |
| Winter | 0.374 ± 0.048 (8) | 0.406 ± 0.018 (8) | 0.359 ± 0.016 (8) |
| *Pectoralis CS* |  |  |  |
| Summer | 181.44 ± 37.10 (5) | 233.28 ± 32.18 (8) | 294.27 ± 35.84 (7) |
| Winter | 220.53 ± 57.48 (7) | 367.65 ± 28.80 (8) | 263.48 ± 49.67 (8) |
| *Heart CS* |  |  |  |
| Summer | 288.88 ± 52.22 (5) | 291.70 ± 30.36 (8) | 312.05 ± 59.09 (7) |
| Winter | 150.67 ± 36.44 (7) | 317.87 ± 31.57 (8) | 203.01 ± 34.13 (8) |
| *Pectoralis HOAD* |  |  |  |
| Summer | 29.49 ± 3.53 (8) | 24.93 ± 3.68^a^ (8) | 40.23 ± 4.11 (7) |
| Winter | 21.75 ± 8.55^a^ (8) | 73.82 ± 6.46^a^ (7) | 36.56 ± 7.43^a^ (6) |
| *Heart HOAD* |  |  |  |
| Summer | 38.19 ± 4.13 (8) | 25.49 ± 1.81^a^ (8) | 41.01 ± 1.74 (7) |
| Winter | 25.18 ± 3.20^a^ (7) | 71.18 ± 3.13^a^ (8) | 43.60 ± 4.32^a^ (8) |

**Table S2**. Mean (± SE) values for pectoralis (one half only), supracoracoideus (one half only) and heart masses (g) and Citrate Synthase (CS) and β-hydroxyacyl Co-A Dehydrogenase (HOAD) activities (μmol min^-1^ g^-1^) for house sparrows after six weeks of acclimation to 24, 5, and -10°C in summer and winter. Sample sizes are included in parentheses. Superscripts refer to significant differences among treatment groups within a season (a).
